# Reducing Epistasis and Pleiotropy Can Avoid the Survival of the Flattest Tragedy

**DOI:** 10.3390/biology13030193

**Published:** 2024-03-17

**Authors:** Priyanka Mehra, Arend Hintze

**Affiliations:** 1 Department for MicroData Analytics, Dalarna University, 791 88 Falun, Sweden; pmh@du.se; 2 BEACON Center for the Study of Evolution in Action, Michigan State University, East Lansing, MI 48824, USA

**Keywords:** epistasis, pleiotropy, mutational robustness, survival of the flattest

## Abstract

**Simple Summary:**

The paper investigates the evolutionary significance of genetic simplicity, proposing that organisms with less complex genetic interactions (low epistasis) and genes with fewer multiple effects (low pleiotropy) possess an evolutionary edge. This hypothesis suggests that such simplicity could lead to greater mutational robustness, allowing these organisms to adapt more efficiently to environmental changes. The experiment demonstrates that these simpler organisms exhibit greater mutational robustness, challenging the traditional belief that complexity is synonymous with evolutionary success. The conclusion highlights the study’s findings that simpler genetic architectures facilitate a higher degree of adaptability and evolutionary success.

**Abstract:**

This study investigates whether reducing epistasis and pleiotropy enhances mutational robustness in evolutionary adaptation, utilizing an indirect encoded model within the “survival of the flattest” (SoF) fitness landscape. By simulating genetic variations and their phenotypic consequences, we explore organisms’ adaptive mechanisms to maintain positions on higher, narrower evolutionary peaks amidst environmental and genetic pressures. Our results reveal that organisms can indeed sustain their advantageous positions by minimizing the complexity of genetic interactions—specifically, by reducing the levels of epistasis and pleiotropy. This finding suggests a counterintuitive strategy for evolutionary stability: simpler genetic architectures, characterized by fewer gene interactions and multifunctional genes, confer a survival advantage by enhancing mutational robustness. This study contributes to our understanding of the genetic underpinnings of adaptability and robustness, challenging traditional views that equate complexity with fitness in dynamic environments.

## 1. Introduction

Evolution optimizes the fitness of organisms within a population over time. We often think of this process as climbing to the peak of an imaginary fitness landscape, and while doing so, organisms move from simpler to more complex forms of life [1]. Although, over time, more and more information related to adaptation to the environment accumulates in genomes [2]. In biology, the term complexity can pertain to different concepts, such as genotypic complexity [3,4], phenotypic complexity [5], developmental complexity [6], or the genotype–phenotype complexity [7,8,9], for example. However, complexity, in general, remains an elusive term and a difficult-to-measure concept [10,11].

It has been argued that changes in complexity mostly happened at the macroevolutionary level [12], which does not exclude that the complexity of organisms within a population can also change in complexity [13] and give rise to macroevolutionary changes later. Here, we focus on the complexity of the genotype–phenotype map and use the degree to which genes interact with the aspect of complexity we study. Consequently, we are addressing changes within a single population given a static fitness landscape. Therefore, we will focus on the complexity of interactions and use the change in these interactions as a proxy to describe the changes in complexity at a microevolutionary level. However, given the right evolutionary circumstances, evolution can also reduce the number of genetic interactions and result in what one might call simpler solutions [14,15]. The question is now about which circumstances give rise to which outcomes. However, before we get to this question, we need to first elaborate on the interactions in the form of epistasis and pleiotropy, explain how robustness can be affected by either, and then explore the role of the fitness landscape.

Mutations introduce a range of genetic variations that can lead to changes in traits known as phenotypic changes. These changes are caused by gains, losses, or modifications to genes [16]. However, these genes do not act in isolation but instead interact with each other [17]. Thus, these interactions, known as epistasis (ϵ) [18], play a crucial role in shaping the ultimate effect of any given mutation. Epistatic interactions can also provide information on how proteins, pathways, and components within cells function together [19]. These interactions explain the complexity of some drug resistance mechanisms [20], improve the understanding of synthetic lethality in cancer treatment [21], and provide insight into the inheritance patterns of complex traits [22].

Positive epistasis plays a crucial role in evolutionary adaptation by facilitating synergistic gene interactions that enhance the organism’s fitness beyond the additive effects of individual mutations. This form of epistasis can significantly contribute to the rapid adaptation of organisms to changing environmental conditions, as it allows for the emergence of complex traits that are highly beneficial for survival [23]. Conversely, negative epistasis introduces a layer of genetic constraint that can limit evolutionary potential. This occurs when the interaction between genes results in a phenotypic outcome that is less advantageous than expected based on the individual effects of the mutations involved. Such antagonistic interactions can hinder the accumulation of beneficial mutations, potentially slowing the pace of adaptation [24].

Unlike epistasis, which involves interactions between different genes, a single gene can also influence multiple traits. This is known as pleiotropy (π) [25]. Pleiotropy demonstrates how a single genetic variation can have wide-ranging effects on an organism’s phenotype, potentially affecting fitness, adaptation, and disease susceptibility. For example, pleiotropic genes have been identified as critical factors in human diseases, where a single gene mutation can lead to multiple symptoms or conditions [26]. Studies on pleiotropy also contribute to the understanding of biological systems, revealing how genes regulate multiple developmental pathways to shape the form and function of an organism [27].

Robustness in biological systems refers to the ability of organisms to maintain the same phenotypic traits despite genetic mutations or variations and environmental challenges [28]. Functional stability is essential to ensure survival and facilitate adaptation. Robustness is achieved through various genetic mechanisms, among which epistasis and pleiotropy play significant roles. For comparison, when a single gene is responsible for a phenotypic trait, when it is hit by the right mutation, that trait disappears, as in the case of antibiotic resistance [29]. However, the effect of such a mutation could be augmented if the same gene is pleiotropic in the sense that it plays multiple roles and thus contributes to multiple traits. A single mutation can have multiple negative effects [25]. Interestingly, this can also go in the other direction; when, for example, a mutation enhances expression, it could benefit multiple traits [25,30]. Mixed situations can be imagined for pleiotropic genes, where a single mutation improves some traits while also preventing others, making pleiotropic genes notoriously hard to study [31].

Conversely, epistasis can also modulate the effect of mutations on single genes. Positive epistasis can lower the effect of a deleterious mutation [23,32], while negative epistasis could enhance the effect. In the extreme case, where many genes have a high degree of epistasis and are pleiotropic, the effects of mutations might become even less predictable and more multifaceted.

Consequently, π and ϵ play a crucial role during evolutionary adaptation but are also the consequence of evolution. However, whether or not an increase or decrease in π or ϵ makes organisms more or less robust is still highly debated [33,34,35,36,37,38]. What likely made answering this question hard is not only the difficulty one faces when doing evolutionary experiments with natural organisms but also the way how computational models were made. In most previous cases related to this matter, evolution occurred at the phenotypic level or did not distinguish between the genotype and the phenotype [39,40]. This is especially curious, as π and ϵ are phenomena that happen in translating a genotype to phenotype. Genes affecting multiple traits or interacting to form a trait can only occur within a model with a genotype and phenotype. This translation from a genotype to phenotype is called encoding within the evolutionary computation community [41] and, of course, the subject of biological research, as we want to know how one can make an organism’s phenotype from its genes.

We previously extended the NK fitness model with such encoding [42]. In this model, a genotype experiences mutations that affect not only the expression of genes but also how they interact epistatically and what pleiotropy a gene has while also resulting in a phenotype with multiple traits. This model uses a simple matrix to determine which gene influences which trait. Natural protein or transcription factor networks are likely more complicated, and thus, other indirect encodings have been used [43,44,45]. However, including further complications from more sophisticated models might induce other confounding findings, and we thus use a very simple method to model encoding. Indirect encodings in nature come from the genotype to phenotype mapping [46], which can be very complicated and thus result in different notions of complexity, such as genotypic, phenotypic, or developmental complexity [47,48], and while interesting, we focus on the relation between the components and how they map to traits in the simplest form and thus are, as described above, considering the number of interactions as a proxy for complexity (interaction complexity), but be aware that the notion of complexity is more nuanced in nature. The question now is in which environment can we test whether π and ϵ increase or decrease to facilitate mutational robustness?

**Figure 1:**
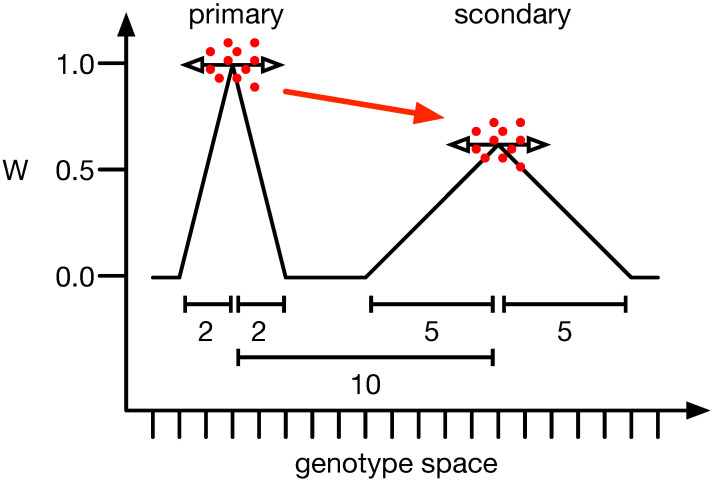
Illustration of the survival of the flattest phenomenon. When comparing the two peaks of different heights, one would expect the population to sit at the highest (primary) and not a lower adjacent one (secondary). However, if the mutation rate and the mutation effect size (horizontal arrows) are strong enough, populations (red dots) converge on the lower one (red arrow). The y-axis illustrates the fitness of all organisms, and the x-axis depicts the mutational distance between genotypes. The scale bars show the mutational distance between peaks (10) or the width of the primary (2) and secondary peak (5).

One of the more interesting examples of mutational robustness and how it influences evolution is the survival of the flattest (SoF) phenomenon. Here, populations with wider genetic variability and flatter fitness landscapes have a greater chance of enduring and adapting in variable environments than those perched on high fitness peaks [39]. Adami et al., 2006 [40] demonstrated that a population might not remain on a high fitness peak, instead preferring a lower one, as long as the lower peak is flatter. In this context, a peak refers to a phenotype with higher fitness than all its mutational neighbors, specifically those with a Hamming distance of 1. Flatness relates to a peak’s average fitness, or neutral network, and describes the mean fitness of these mutational neighbors (see Figure 1). If the mutation rate is high enough [49], populations are small [39], or the ratio between the primary and secondary peaks is closer to 1.0 [39,49], populations will prefer the secondary peak.

This SoF landscape also presents an interesting challenge to adaptation. The higher and narrower peak can only be reached or remained on by a population with higher mutational robustness against the mutation rate. Previous studies clearly showed that reducing the mutation rate is one way un which the population can stay at a higher peak. Alternatively, making the landscape around the higher peak wider or the flatter peak smaller was modeling mutational robustness, and both could control the experiment’s outcome. Previous studies considered only computational models in which there was no distinction between genotype and phenotype. Thus, a change in the fitness landscape did not change the way the genotype translates to the phenotype in any way, as both were the same in these models. Here, instead, we use a model with a genotype-to-phenotype translation and the ability to evolve π and ϵ, thus allowing us to investigate how both can facilitate or hamper mutational robustness.

We will show that computational model organisms having an indirect encoding from genotype to phenotype experience the same survival as the flattest phenomenon as directly encoded ones. However, in indirectly encoded organisms, mutational robustness is determined by the effect that mutations have on the individual genes and how they interact, specifically their degree of epistasis and pleiotropy. The question we answer here, which has been debated before, is whether an increase or decrease in epistasis or pleiotropy increases or decreases mutational robustness. We show that a reduction in both epistasis and pleiotropy makes organisms more mutationally robust and thus allows them to avoid the survival of the flattest tragedy of not being able to remain at a higher, narrower peak. It is essential to note that the survival of the flattest phenomenon is about the ability of a population to remain at a higher peak or about two populations in competition ending with the slower replicating one outcompeting the one at the higher peak due to mutational robustness. Therefore, we focus on epistasis and pleiotropy’s effect on mutational robustness and a population’s ability to maintain their position at the higher peak, and while that does not exclude a way for a population to climb to a higher peak by reducing its mutational robustness, our work does not seek to find evidence for the latter. We, however, argue that such a reduction in epistasis and pleiotropy reduces the number of interactions, makes organisms less genetically *complex*, and thus presents another example where evolution prefers a simpler over a more complicated solution.

## 2. Materials and Methods

### 2.1. Indirect Encoding

**Figure 2:**
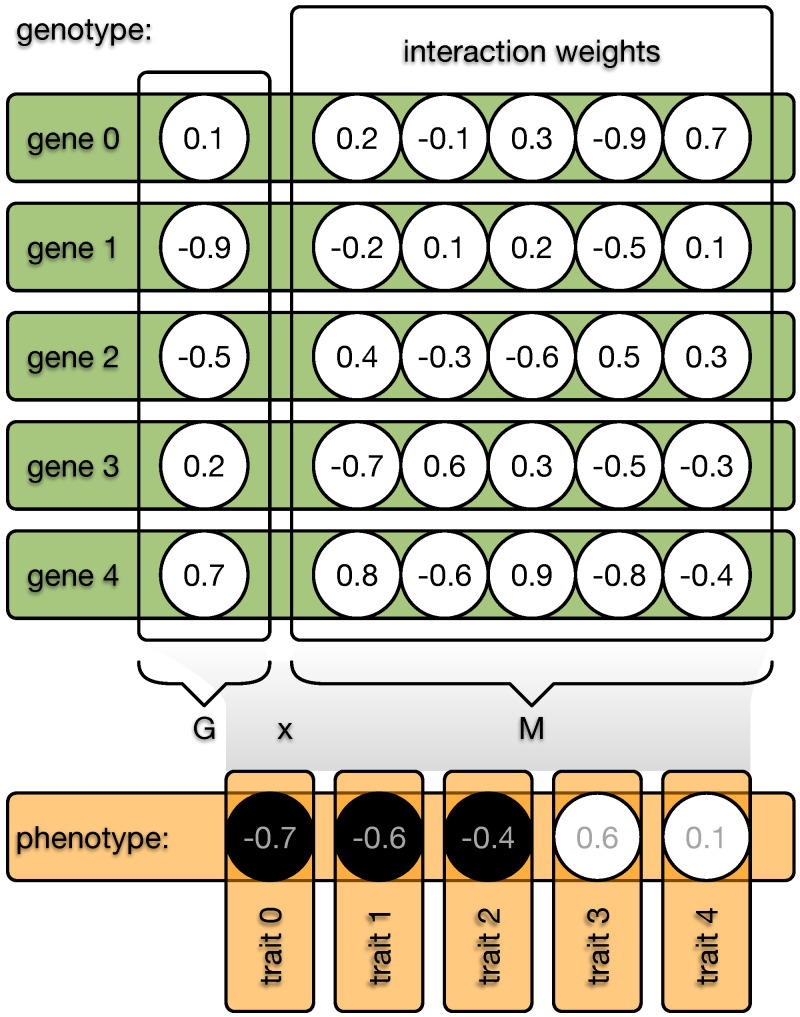
Illustration of the indirect encoding method. Each organism possesses a genome made from *N* genes. Each gene (green bar) is defined by a value and a vector of length *N* (circles). These gene vectors form the interaction matrix *W*, and the values of each gene form the vector *G*. The dot product between G and M results in the phenotype vector (orange), which becomes discretized (black or white circles) and each element defines one of the *N* binary traits of the phenotype. Mutations can now change all values in the genome, and the interaction between G and M can indirectly affect the phenotype. In the case of direct encoding, *W* would be an immutable identity matrix.

The indirect encoding method distinguishes between an organism’s genotype and phenotype. The genotype is organized into *N* genes, each with an expression value—imagine a catalytic activity, for example—in the range of [−1.0,1.0]. Each gene can interact with all other *N* genes, including itself, and these interaction weights are defined by a value from the range [−1.0,1.0] (see Figure 2). The phenotype can be computed by taking the dot product between the expression values *G* and the interaction weights *M*. The resulting phenotypic vector *P* is then discretized so that all positive values become 1, while all other values become 0. This vector, of size *N*, is then treated as the traits of the classic NK model, and the fitness can be assigned depending on *K*. However, the degree to which genes can interact is now freely evolvable by changing the expression values *G* and the weights of the interaction matrix *M*, independent of *K*.

### 2.2. Epistasis and Pleiotropy

In our extended model [42], the degree of genetic interactions determining traits is decoupled, while those interactions can still evolve. Therefore, we need to independently measure epistasis (ϵ) and pleiotropy (π).

First, we calculate an interaction matrix (IM) that reveals how individual genes control individual traits. Since each organism has *N* genes and *N* traits, the IM matrix is square, with dimensions N×N [42]. To test if a gene affects a trait, its expression value $G_{i}$ is set to −1.0 and 1.0. For both alternatives, the phenotype is constructed (P=G×M), and for each trait, $P_{j}$, it is determined if the change in gene *i* alters trait *j*. If so, the interaction matrix $IM_{i,j}$ becomes 1; otherwise, it becomes 0. The sum of each row of the matrix shows the number of traits influenced by each gene (π), while the sum of each column shows the number of genes contributing to each trait (ϵ). The mapping of genes to traits relies on the mapping matrix, which evolves. Because mutations appear randomly, the resulting vectors for π and ϵ of two organisms cannot be compared directly. To address this, we sort the vectors from low to high [42], which ignores the exact ϵ or π of each individual gene, but instead provides a distribution over the entire organism.

**Figure 3:**
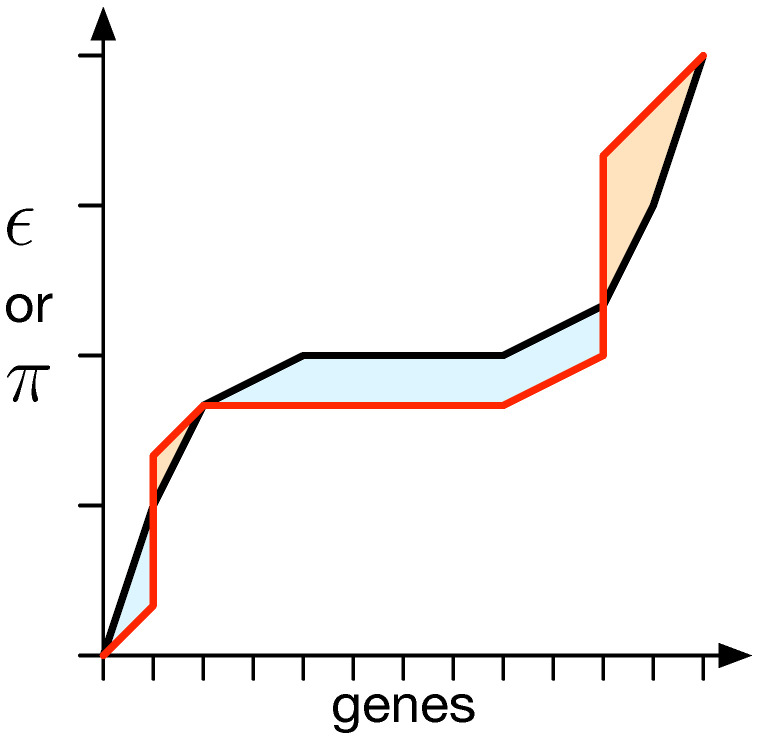
Difference between the expected distribution of epistasis (ϵ) or pleiotropy (π) and a specific measurement. For all genes, their degree of ϵ or π, depending on what is supposed to be measured, is obtained, and the values are sorted in an ascending manner. This results in a specific organism’s value distribution (red line). This needs to be compared to the random expectation (black line). Thus, the surfaces between both lines (red and black) that are above (*a*) the random expectation, shown in orange, and their negative counterparts below (*b*) the expectation, shown in blue, are measured. The total difference is then determined to be a−b. In the vast majority of measurements taken, the measurements illustrated as the red line do not straddle above and below the expectation but are strictly on either side. This method, however, takes care of intermittent cases where values end up not being strictly above or below.

We used a method to quantify the difference between ϵ and π from the random expectation (black line; see Figure 3). The random expectation is determined using a 1000 mapping matrix with values drawn from a uniform distribution [−1,1]. This method yields a negative value if ϵ and π are lower than the random expectation and a positive value otherwise [42].

We compute the area between the measured ϵ or π and the random expectation to quantify the difference between them. The total area above the random expectation is calculated independently of the area below it, resulting in two measurements, denoted as *a* for above and *b* for below. The difference between the random expectation and the measured ϵ or π is then quantified as the distance Δ, which is equal to the difference between *a* and *b* (see Figure 3).

### 2.3. Two-Peak Model

The computational model [50] to test the survival of the flattest phenomenon is almost identical to the one used by Channon et al., 2011, which itself follows the experimental design of Wilke 2001 and Comas 2005 [39,51]. In this landscape (see Figure 1 for an illustration), the traits selected are a binary vector of length N=20. However, there are only two peaks exactly ten mutations away from each other. The primary peak has a fitness of one, with a mutational neighborhood with a Hamming distance of 1, with each genotype having a lower fitness. When not explicitly specified, that fitness is 0.5. The height of the secondary peak ($W_{sec}$) is lower depending on the conditions tested (here 0.6, 0.7, 0.8, or 0.9). The mutational neighborhood includes up to four more mutations. Their height depends on their Hamming distance linearly dropping (Hamming distance of 1 has a fitness of $0.8W_{sec}$, distance 2 $0.6W_{sec}$, distance 3 $0.4W_{sec}$, and finally distance 4 $0.2W_{sec}$). All other genotypes have a fitness of 0.0.

The main difference between the two-peak model used here and the predecessors [39,50,51] is indirect encoding; while fitness is still calculated based on the organism’s traits defined by its phenotype, indirectly encoded organisms have a genome that determines the phenotype, and while the location of a peak defines the phenotype, it does not define the genotype. Thus, here, two random organisms with an indirect encoding are generated until we find a pair whose phenotype has a Hamming distance of 10. Then, the fitness values for the peaks and surrounding mutations are defined.

In this experiment, half of the population starts at the primary peak, and the other half starts at the secondary peak. This is carried out by generating a random organism and using its phenotype to define where the highest peak is. Then, organisms are generated at random until one is found that has exactly ten phenotypic traits different than the first, which is used to define the center of the second peak. This method is necessary to prevent organisms from evolving all their sites to 0. In the indirect encoded model, if all elements in the mapping matrix become negative or 0.0, the resulting phenotype is also all 0 with no interaction between genes. This does not imply that the trait in question is removed, like the loss of a phenotypic feature. It means that this trait now has a value of 0 instead of 1. Similarly, traits are not added when their value becomes 1 which is a limitation of this computational model system [48]. Instead, in this static fitness landscape, we have N=20 traits in the state of 0 or 1. One could think of these traits as the white or black color of the peppered moth *Biston betularia* [52], which evolves from being white to black as a response to the pollution caused by the Industrial Revolution. Here, white would be the absence or low expression of a pigment (lack of interactions), while the black phenotype would require the presence or upregulated expression of a pigment (presence of interactions).

Thus, a genotype of all 0 could be adaptive when either of the peaks or their respective vicinity has that phenotype. However, since we first pick a random genotype, which has on average half of its phenotypic sites 0 and the other 1, and the secondary peak to have 50% of these sites flipped from 0 to 1 or 1 to 0, we ensure that at least five phenotypic sites have to change from 0 to 1 (on average), which can not be achieved by just removing all interactions. Even if, by chance, the second peak is accidentally at the phenotype made from all 0, it would be not only an infrequent occurrence (with a chance of $9.54\times 10^{-7}$ considering 20 times the same draw from a binomial distribution with p=0.5), but it would also happen into the other direction in half of the cases. In other words, the selection of peaks for the high number of samples, and the juxtaposition of both peaks to be ten mutations different from each other, prevents adaptation from just removing all interactions to achieve high fitness.

Like in the previous models, organisms can evolve for 10,000 generations, given a specific mutation rate μ and population size. It was recorded if, within this time, either of the peaks was vacated, indicating that a population either lost both or converged on the other peak. A peak is considered vacated when no individuals are present anywhere in its mutational range. This experiment was repeated 10,000 times. Given the times at which the primary $P_{1}$ and secondary $P_{2}$ peaks were vacated, we can calculate the fraction of times that the population remained at the highest peak:

(1) $$p\left(P_{1}\right)=\frac{P_{1}}{P_{1}+P_{2}+1}$$

In Equation (1), a pseudo count of 1 accounts for very high mutation rates in which neither peak remains populated. This approach allows us to examine the dynamics of the survival of the flattest phenomenon and understand how the population navigates the rugged fitness landscape under different mutation rates and population sizes. The results can then be analyzed to determine the influence of the indirect encoding and the adaptability of mutational robustness on the population’s ability to avoid the survival of the flattest phenomenon and find higher fitness peaks.

### 2.4. Changing Epistasis and Pleiotropy while Keeping the Phenotype Identical

The indirect encoding method provides a unique opportunity to study how different genotypes result in the same phenotype vector, allowing for an exploration of the effects of varying epistatic and pleiotropic interactions on mutational robustness. By manipulating the gene expression values *G* and interaction weights *M* while keeping the phenotype constant, we can observe the consequences of changes in ϵ and π on an organism’s adaptability and survival.

To study these effects, we implement a Hill climber algorithm that compares an organism with its mutant counterpart, which has the same phenotype but different degrees of ϵ or π. Depending on our goal, we can either maximize or minimize these values and observe the resulting changes in mutational robustness. We can also simultaneously manipulate ϵ and π or choose one randomly to emulate drift.

In this experiment, we generate a random mutant with μ=0.001 until we find one with an identical phenotype. If the mutant has a different degree of ϵ or π, it is retained, given the direction in which ϵ or π should be changed: higher or lower. The selection process is then repeated 100 to 1000 times. Throughout this process, the phenotype remains constant, ensuring that the organism’s position in the fitness landscape is unchanged. However, the values of ϵ and π can be altered, allowing us to observe the impact of these changes on mutational robustness.

## 3. Results

The two-peak model has previously been tested only on directly encoded organisms [50,51]. It demonstrated that, in the absence of the capacity to evolve mutational robustness, the probability of staying at the higher peak given the mutation rate depends on three factors: the height of the second peak, population size, and the flatness of the first peak. Consequently, we expect the same parameters to control the adaptation of indirectly encoded organisms. Namely, lower mutation rates or lower secondary peak heights allow populations to stay on the higher peak, and smaller population sizes also cause populations to leave earlier.

**Figure 4:**
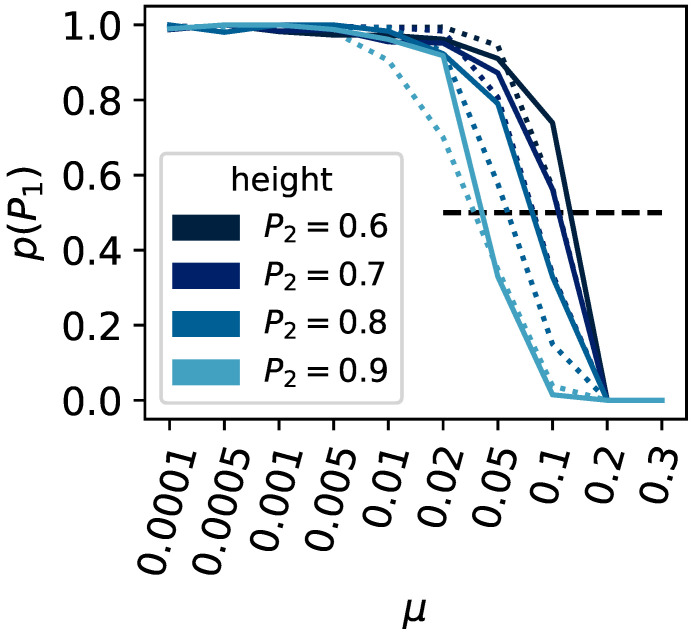
Survival of the fittest versus flattest. The x-axis shows the mutation rate μ, and the y-axis shows the ratio of experimental runs that remained on the highest fitness peak $P_{1}$ compared to all experiments that did not stay on either peak $P_{1}$ or $P_{2}$. The population size was varied, and results shown as a solid line indicate a population size of N=1000, dashed for N=100. The height of the second peak was varied (0.9, 0.8, 0.7, and 0.6); see the legend for the corresponding colors.

Upon varying the height of the second peak (0.6, 0.7, 0.8, and 0.9) or the population size (ranging from 100 to 1000), we find our expectations corroborated (see Figure 4). The critical mutation rate, at which a population can no longer remain at the highest peak, decreases as the secondary peak becomes taller. Likewise, larger populations can withstand a higher critical mutation rate before they cannot stay at the higher peak.

**Figure 5:**
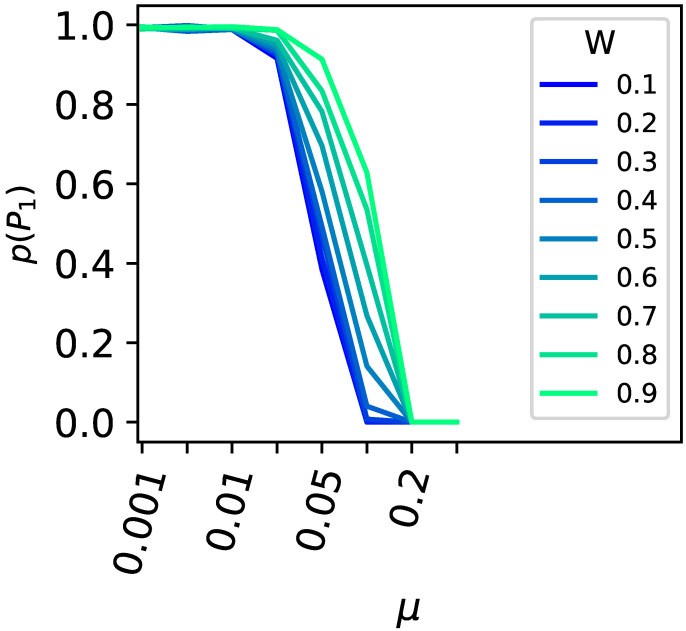
Survival of the flattest versus the drop in fitness surrounding the higher peak. All one-mutant neighbors around the higher primary peak were set to different fitness values (0.1 to 0.9 in 0.1 increments; see legend for the color code), and the likelihood for the population to remain at the primary peak (y-axis) was plotted against the different mutation rates (x-axis). Populations of size 100 were tested in 10,000-replicate experiments.

The critical mutation rate for a population to leave a peak is also influenced by the direct mutational environment of the higher peak. The higher the fitness of the mutants surrounding the highest peak, the greater the critical mutation rate must be for the population to vacate the peak. This effect is corroborated in the two-peak model using indirect encoding (see Figure 5).

These results confirm that applying the two-peak model to indirectly encoded organisms, which can potentially evolve mutational robustness and vary in their ϵ and π, produces the same observations regarding the survival of the flattest phenomenon.

We observed that organisms adapt to more rugged adaptive landscapes (where genotype-to-phenotype relationships are more complex) by reducing ϵ and π [42]. We hypothesize that the reduction in ϵ and π causes organisms to experience the survival of the flattest phenomenon less frequently. We propose that to have a chance of staying at a higher peak, organisms increase their mutational robustness by decreasing ϵ and π. It is known that ϵ is correlated with mutational robustness [49].

Consequently, we predict that organisms should experience survival of the flattest frequently with a higher degree of ϵ. We believe that π plays a similar role, with a gene having more interactions being less robust to mutations. Therefore, a higher degree of π should also lead to organisms experiencing the survival of the flattest effect more often.

**Figure 6:**
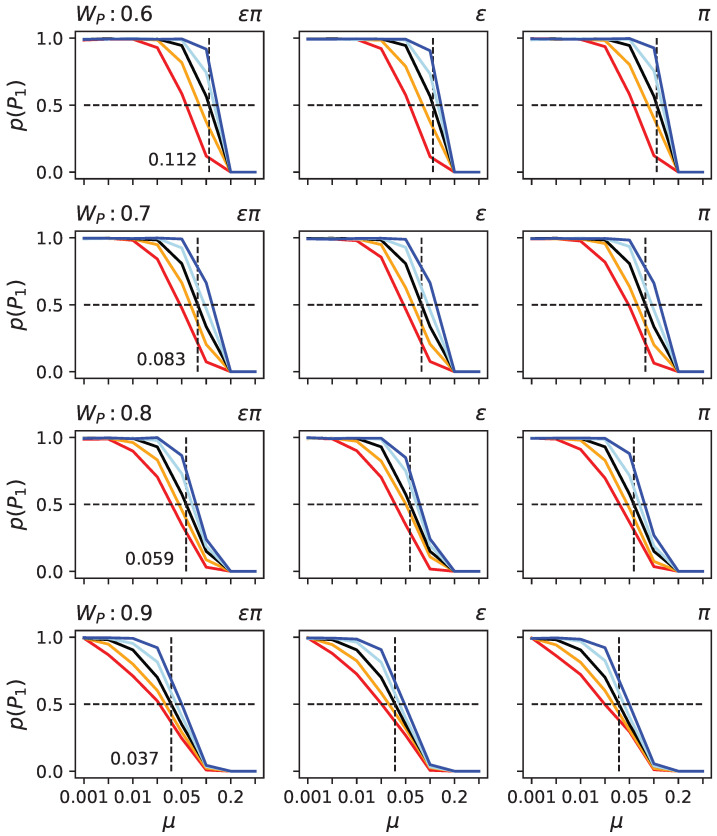
Probabilities for populations to remain at the primary peak under various conditions in the two-peak model. Each row depicts results for different heights of the secondary peak ($W_{P}$: 0.6, 0.7, 0.8, and 0.9). In each sub-panel, the black line indicates the likelihood of remaining at the primary peak (y-axis) given the mutation rate μ (x-axis). The other colors (blue, light blue, orange, and red) indicate that the organism first experienced different rounds of bias to ϵπ (**left** column), ϵ (**middle** column), or π (**right** column). Light blue indicates 100 rounds of decrease, in dark blue, 1000 rounds, in orange, 100 rounds of increase, and in red, 1000 rounds of increase. Since changing the product of ϵ and π has almost identical effects to changing ϵ or π independently, the columns appear to be very similar. They are thus given as a confirmation for that it does not illustrate a difference.

To test these hypotheses, we took randomly generated organisms with indirect encoding. We determined the critical mutation rate at which they could not remain on the primary peak in the two-peak landscape given the height of the secondary peak (see Figure 6 black line). As expected, the higher the secondary peak, the lower the critical mutation rate at which populations vacate the primary peak.

To assess the effect of ϵ and π, we took the same randomly generated organisms. Still, before exposing them to the two-peak landscape, we artificially increased or decreased their levels of ϵ and π using a hill climber (see Materials and Methods). Observe that the hill climber is not supposed to model an evolutionary process; it is only used to alter ϵ and π so we can test those organisms in the SOF landscape. We found that, as predicted, organisms with increased ϵ or π have a lower critical mutation rate, while decreased ϵ and π result in a higher critical mutation rate (see Figure 6). Moreover, the critical mutation rate also depends on how much ϵ and π were modified. Finally, the effect of altering ϵ and π together, or each individually, is nearly indistinguishable, suggesting that a change in one causes a change in the other in the same direction. Such behavior might be explained by the relationship between genes and traits. If we consider a functional matrix that maps a column of components to a row of functions, we can define epistasis for a trait as the sum of a column and pleiotropy as the sum of a row. If we were to alter that matrix, such as by removing a connection between a gene and a trait, then both ϵ and π would be reduced. This concept applies to any functional mapping matrix and is not reliant on a specific model. We consequently believe that this constraint affects ϵ and π in the same manner.

These findings suggest that organisms could endure the survival of the fittest phenomenon for more extended periods if they decreased ϵ and π.

## 4. Discussion

Incorporating indirect encoding into our evolutionary model maintains phenotypic selection pressures while introducing a new dimension to the fitness landscape through genotypic mutations. Unlike indirect encoding, where genotypic alterations directly translate to phenotypic changes, the indirect approach introduces a broader array of potential genotypic variations, especially epistatic and pleiotropic effects. This change in the interaction complexity alters the fitness landscape, yet our findings reveal that the SoF principle can still be observed. Indirectly encoded organisms face challenges in sustaining themselves on the evolutionary peak, especially as mutation rates climb, population sizes diminish, or the secondary peak’s prominence increases. Thus, mutational robustness is still present, and therefore, the SoF landscape allows us to investigate how epistasis and pleiotropy facilitate said robustness.

Our analysis demonstrates that organisms exhibiting lower levels of epistasis and pleiotropy exhibit enhanced mutational robustness, stabilizing their position at the highest peak. This insight underscores the intricate balance between genetic architecture and evolutionary adaptability, highlighting how the configuration of genotypic to phenotypic mappings can significantly influence an organism’s evolutionary trajectory.

While our computational model and the concept of indirect encoding serve as abstract representations of genetic interactions, they offer valuable insights that may align with biological realities. The model suggests that organisms characterized by lower levels of π and ϵ exhibit greater mutational robustness—a hypothesis that merits empirical investigation in natural settings. Similarly, more complicated models incorporating indirect encoding, such as gene regulatory networks, should be tested for the same effect. While we expect the same phenomenon to occur qualitatively, we also expect quantitative differences. However, our proposition aligns with the broader scientific inquiry into genetic robustness and its evolutionary implications, suggesting that similar principles could underpin both computational and biological systems.

Moreover, our findings contribute to the ongoing debate about complexity in biological systems, specifically the complexity of interactions between genes and traits. Contrary to the prevailing notion that complex environments necessitate complex genetic architectures, our model presents a counter-narrative. It illustrates that simplicity, regarding reduced genetic interactions and functionalities, can confer a survival advantage in certain evolutionary landscapes.

While our results show that organisms can remain at high and narrow peaks by reducing epistatic interactions and pleiotropy of genes to increase mutational robustness, it falls short of elucidating the evolutionary journey from a lower to a higher peak. The SoF landscape is characterized by a vast fitness valley between the lower (ancestral) peak and the higher one. Valley crossing mutations are required to traverse this landscape to transition from a lower, broader peak to a higher, narrower one. However, we have not studied this phenomenon here.

Still, we can imagine a dynamic landscape where peaks evolve—growing higher and narrower—suggesting that organisms must adapt by honing their mutational robustness to secure their position atop the increasingly precarious peak, thereby avoiding descent into less optimal fitness zones. Our study demonstrates that adaptation, in terms of retaining a position on the higher peak, can be achieved by reducing π and ϵ. However, this mechanism’s role in the ascent of the fitness landscape needs to be verified using alternate fitness models. This exploration underscores the interplay between genetic architecture and evolutionary dynamics. Future research, perhaps utilizing models with temporally varying landscapes, is crucial to unravel these dynamics further.

## 5. Conclusions

Our investigation into the evolutionary dynamics within the framework of indirect encoding has shed light on the interplay between genetic architecture and evolutionary adaptability. In the survival of the flattest (SoF) fitness landscape, evolving organisms struggle to remain at the highest peak as their vulnerability to mutations drives them towards a flatter and broader peak. We have shown that reducing epistasis and pleiotropy levels enhances mutational robustness, enabling organisms to maintain their foothold on the higher peak. This insight is crucial, suggesting that simplicity in genetic interactions may be a favored evolutionary strategy under the conditions exemplified here. Moreover, this work contributes to the broader discourse on biological complexity, challenging the notion that complexity is a prerequisite for adaptation and survival within an evolving population.

However, the hypothesis that organisms with lower π and ϵ levels exhibit greater mutational robustness beckons further investigation in natural settings, while we have demonstrated the potential for adaptation by reducing genetic interactions, the evolutionary pathways leading from lower to higher peaks—especially across vast fitness valleys—remain unexplored within our current model. Future research, potentially employing models with temporally evolving landscapes, will be essential to unravel the role of mutational robustness in evolutionary adaptation fully.

## Data Availability

All data and code will be made available through GitHub upon acceptance of the manuscript for publication.

## Abbreviations

The following abbreviations are used in this manuscript:

SoF	Survival of the Flattest
ϵ	Epistasis
π	Pleiotropy
